# Determinants of mobile money loan disbursements: Evidence from Uganda’s post pandemic digital credit boom

**DOI:** 10.1371/journal.pone.0338535

**Published:** 2026-07-01

**Authors:** Lorna Katusiime, Frank W. Agbola

**Affiliations:** 1 Monetary Policy Department, Bank of Uganda, Kampala, Uganda; 2 Newcastle Business School, The University of Newcastle, Callaghan, New South Wales, Australia; Universiti Malaysia Sabah, MALAYSIA

## Abstract

In the aftermath of the COVID-19 pandemic, Uganda witnessed a rapid rise in mobile money usage and digital credit adoption, underscoring the sector’s role in post crisis recovery and financial resilience. Against this backdrop, this paper examines the determinants of mobile money loan disbursements in Uganda a global pioneer in mobile financial innovation, using monthly data from July 2021 to December 2024 and applying the Autoregressive Distributed Lag (ARDL) cointegration framework to capture both long-run relationships and short-run dynamics. Results show a long-run relationship linking average loan disbursements with outstanding loan values and lagged inflation. In the short term, past loan disbursements have a significant impact on current loan values. Behavioural proxies are informative, airtime purchases and timely mobile loan repayments are associated with higher disbursement volumes, consistent with lenders interpreting them as signals of reliability and liquidity. By contrast, higher transaction volumes and fees depress disbursements, underscoring the adverse impact of elevated user costs on credit access. These findings highlight actionable levers for expanding responsible digital credit and deepening inclusion.

## Introduction

Financial exclusion remains one of Sub-Saharan Africa’s (SSA) most persistent development constraints. The region has the highest share of unbanked adults globally, with over 40 percent lacking access to formal financial services [1]. This exclusion limits their ability to save securely, smooth consumption during economic shocks, and undertake productive investments and highlights the critical roles of financial inclusion for supporting economic growth and improving livelihoods [2,3]. Traditional financial institutions have struggled to reach low income and rural populations due to high operating costs, limited physical infrastructure, and the absence of reliable credit information [1,4]. However, the diffusion of mobile money and other digital financial services (DFS) has transformed this landscape [1,4,5].

A key element of this transformation is the rise of digital credit, instant, small value loans disbursed and repaid through mobile channels, which has become central to the region’s financial inclusion strategy. As mobile phone ownership and access to mobile financial services have expanded, the range of features offered through mobile money platforms has also increased, including the ability for account holders to borrow [1,4]. In several Sub-Saharan African economies, mobile money accounts have emerged as the dominant source of formal credit [1]. While digital credit has widened liquidity access for marginalised households and micro-enterprises, its rapid growth has generated complex challenges. For borrowers, high pricing, short repayment cycles, and automatic wallet deductions may heighten affordability and over indebtedness risks. For lenders, compressed risk assessment horizons, lack of collateral, network outages, and regulatory uncertainty may add liquidity and operational stress. These pressures make loan disbursement decisions, how much to lend, to whom, and when, a critical margin through which inclusion, profitability, and stability interact. Additionally, prevailing macroeconomic conditions are a critical consideration, as they significantly influence credit demand, lending behaviour, and overall market dynamics. For both lenders and borrowers, fluctuations in inflation, interest rates, and economic activity directly influence credit demand, lending decisions, and overall market performance.

Although mobile money has attracted widespread attention as a catalyst for financial inclusion, particularly in developing economies, empirical evidence on the factors that drive mobile network operators’ (MNOs) engagement in credit provision remains limited. Understanding the determinants of mobile money loan disbursements is critical for designing effective policies that harness digital finance to promote financial inclusion and growth. This study addresses this gap by empirically examining the drivers of mobile money loan disbursements by MNOs in Uganda. Accordingly, this study provides novel evidence and insights into the operational and macroeconomic factors shaping digital credit dynamics in Uganda and, by extension, contributes to the broader understanding of mobile credit markets across Sub-Saharan Africa. Uganda offers a particularly compelling case for such an inquiry as one of the earliest adopters of mobile money technology globally [6], where mobile money has gained remarkable traction over traditional banking, with 65.9 percent of Ugandans using mobile money services compared to 13.7 percent who use commercial banks [7] and mobile based loans becoming a mainstream source of short term credit [1]. To the best of our knowledge, it is the first study to analyse the determinants of MNO led mobile money loan disbursements in Sub-Saharan Africa, offering new insights into the dynamics underpinning digital credit provision

Using monthly data from July 2021 to December 2024, this study applies the Autoregressive Distributed Lag (ARDL) cointegration framework of Pesaran et al. [8] to examine the determinants of mobile money loan disbursements in Uganda. Covering a period of post pandemic recovery, the analysis investigates how institutional and macroeconomic factors jointly influence the value of average mobile loan disbursements and seeks to identify the main drivers behind variations in mobile money lending activity. Results reveal a long run relationship between mobile loan disbursements, outstanding loan value, and lagged inflation. In the short run, previous loan disbursements exert a strong influence on current lending activity. Guided by signalling theory [9], the analysis interprets airtime expenditure and repayment timeliness as behavioural signals that lenders use to infer borrower reliability in the absence of formal credit histories. The study finds behavioural indicators, such as increased airtime purchases and timely loan repayments, are positively associated with higher disbursement values, reflecting lenders’ interpretation of these behaviours as signals of borrower reliability and liquidity. Conversely, greater transaction volumes and higher service fees tend to dampen disbursements, highlighting the constraining effect of elevated user costs on access to digital credit. These findings provide the first empirical evidence on the institutional and macroeconomic determinants of MNO led mobile money loan disbursements in Uganda, offering new insights into the dynamics of digital-credit markets in Sub-Saharan Africa.

The rest of the paper is organised as follows. Section 2 provides an overview of the literature on mobile money. Section 3 describes the mobile money market in Uganda. Section 4 discusses the methodology and estimation technique employed in the analyses. Section 5 reports and discusses the empirical results. Section 6 concludes with policy recommendations.

## Literature overview

Financial inclusion is a key driver of economic growth [2,3], and the rapid diffusion of mobile money technology has emerged as a powerful tool for extending financial access, particularly in developing economies [10–12]. Mobile-based lending is an innovative and rapidly growing trend in the financial sector that enables individuals to access loans through mobile platforms, where the entire process, from loan application to disbursement, takes place on a mobile device without requiring a traditional bank account [13,14]. This form of lending has become especially important in low-income countries, where access to conventional banking services is limited, leaving many individuals without credit options [13–15]. However, it differs fundamentally in its screening technology, product design, and operational model. Rather than relying mainly on collateral, documented income, and credit histories, digital lenders frequently employ alternative data, including mobile money transaction histories, call detail records, airtime patterns, handset indicators, and behavioral usage to estimate repayment likelihood using automated or algorithmic scoring models [12–16]. These technologies allow the provision of small value, short tenor loans to users with little or no credit history and enable credit limits and pricing to be updated dynamically as new behavioral and transactional data arrive.

This algorithmic and transaction based design of digital credit scoring models implies that the determinants of mobile money loan disbursement may be structurally different from those identified in the traditional banking literature. In digital ecosystems, eligibility, loan size, and the timing of disbursement are often functions of platform signals such as transaction intensity, regularity, repayment performance, liquidity flows, and user engagement. Consequently, mobile money activity itself becomes central to credit supply, because it simultaneously signals a user’s repayment capacity and the platform’s ability to fund lending through the liquidity circulating within the mobile money ecosystem. Evidence from digital credit scoring studies supports this mechanism where behavioural patterns derived from mobile phone and transaction data can predict repayment performance and may perform competitively relative to traditional approaches, particularly where formal credit histories are limited [16]. This literature motivates treating variables such as transaction volumes, agent liquidity, and customer balances not as peripheral controls, but as core determinants of disbursement within mobile money lending models.

Beyond underwriting, recent digital credit literature has focused on several areas that are directly relevant for interpreting loan disbursement dynamics. First, empirical literature suggests alternative data and automated screening expand access to previously excluded borrowers, reshaping the information environment and lowering origination costs [16]. Second, impact based studies examine how digital loans support liquidity management and consumption smoothing for marginalised groups who are often excluded from formal financial systems, especially during periods of economic stress, by providing rapid credit that can be used to respond to shocks [17]. This is particularly relevant after COVID-19, which accelerated the adoption of digital financial services and changed household liquidity needs and payment behaviour. Third, policy and market conduct literature highlights that rapid, low friction digital borrowing can also produce risks, including repeat borrowing, short repayment cycles, and over indebtedness, especially when product terms are opaque or borrowers have limited financial capability [18–20]. Wamalwa [18], for instance, links digital credit use and household indebtedness to financial literacy dynamics, underscoring that repeated borrowing can become routine in ways that may not be captured by conventional credit market models. These findings imply that observed disbursement surges may reflect not only expanded access, but also behavioural dynamics such as frequent rollover borrowing and cost insensitive demand, with potential feedback effects on platform underwriting and credit limit updates.

Another digital finance specific dimension concerns the governance and implications of algorithmic scoring. Because digital lenders embed automated decision rules into credit allocation, the determinants of disbursement are shaped not only by macroeconomic conditions but also by the design of scoring models and their data inputs. Algorithmic scoring introduces new trade offs involving efficiency, fairness, transparency, and privacy, which can affect who receives credit and at what limits even when borrowers share similar economic fundamentals [21]. Related regulatory scholarship and policy discussions in East Africa emphasize that market conduct and consumer protection frameworks can influence lender practices, borrower outcomes, and ultimately the trajectory of digital credit expansion, especially in fast growing markets [22].

Despite this growing body of evidence on alternative data scoring, borrower outcomes, and consumer risks, much of which is concentrated in Kenya, there is a paucity of empirical research on the determinants of mobile money loan disbursement from the perspective of mobile money ecosystems and mobile network operators. Previous works have explored how fintech credit affects bank performance, competition, and growth [23–27], yet they stop short of explaining the drivers of loan supply decisions within mobile money ecosystems. Moreover, the literature on mobile money credit remains heavily skewed toward two themes: first, the impact of fintech credit, including mobile lending, on traditional financial markets, particularly the banking sector, and second, the factors that shape the development and adoption of mobile money credit, often employing qualitative approaches to understand users’ perceptions [28].

The survey by Ahmad et el. [12] identifies several areas where further empirical investigation is urgently needed on mobile money and digital credit. Indeed studies examining the determinants of mobile money lending remain scarce and given this paucity of evidence, the present study draws on the well established literature on traditional bank lending to inform its theoretical framing and empirical approach, discussed in the subsequent section on methodology. Studies on conventional banking systems provide a useful benchmark for understanding credit supply dynamics. In Ethiopia, Birhanu et al. [29] found that deposit size, credit risk, portfolio investment, average lending rate, real GDP, and inflation positively affected bank lending, while liquidity ratios constrained it. Similarly, Gebreezgi [30] examined factors influencing loan disbursement of commercial banks in Ethiopia and found that profit, capital and the number of customers positively affected loan disbursement, while factors like deposits, assets, liabilities and the number of branches had no statistically significant effect on loan disbursement. In Nigeria, Olokoyo [31] demonstrated that both institutional factors, such as deposit size and liquidity, and macroeconomic variables, such as economic growth, interest rates, reserve requirements, and foreign exchange rates, significantly influence lending behaviour. The study further noted that commercial bank deposits have the greatest impact on lending behaviour. Olumuyiwa [32] examined the drivers of investment portfolios and lending rates and concluded that loans and advances were positively associated with deposit volume, the annual exchange rate of the Naira to the dollar, GDP, and cash reserve ratio, but negatively related to investment portfolios and lending interest rates. Together, these studies affirm that both institutional capacity and macroeconomic stability are central to understanding loan disbursements, offering a conceptual foundation for examining digital credit.

Emerging evidence on fintech and mobile lending markets suggests that both demand and supply side factors drive credit expansion, though this study focuses exclusively on the supply side. Economic conditions significantly impact the development of digital lending services [33]. However, the effect of economic growth and development on mobile lending is mixed, with some studies finding that it can promote the growth of mobile lending by enhancing borrowers’ ability to repay loans and increasing the funding available to mobile lenders. Other researchers have suggested that in more developed economies, the relationship between economic development and mobile lending weakens or even turns slightly negative [33,34]. Financial regulation and institutional characteristics also play a defining role, stringent frameworks can restrict fintech participation, whereas conducive regulatory settings foster innovation and credit outreach [33]. Moreover, the effect of financial development appears asymmetric in that while it often stimulates consumer lending, it can dampen business-oriented credit [28,34].

Empirical studies that directly assess the determinants of mobile money loan disbursements, particularly from the perspective of mobile network operators (MNOs), remain virtually absent, especially in low income settings where digital finance has outpaced formal banking. This study addresses that empirical void by investigating the determinants of mobile money loan disbursements in Uganda during the post pandemic digital credit boom, one of the earliest and most dynamic mobile money markets globally. Drawing from traditional banking theory and adapting it to digital credit contexts, the study examines how key institutional and macroeconomic variables, including inflation, interest rates, economic activity, transaction volumes, agent balances, and customer liquidity, shape MNO lending behaviour. By focusing on the supply side mechanics of digital credit, the research contributes novel evidence on how mobile money operators allocate credit in a rapidly evolving financial landscape. Moreover, the post pandemic period is likely to be structurally distinct because changes in payment behaviour, liquidity stress, adoption intensity, and macro financial conditions may interact with platform based credit scoring in ways not captured by existing studies. The findings are expected to inform both digital lending strategies and policy frameworks aimed at enhancing financial stability, inclusion, and market efficiency in frontier economies.

## Overview of the Ugandan mobile money market

**Fig 1:**
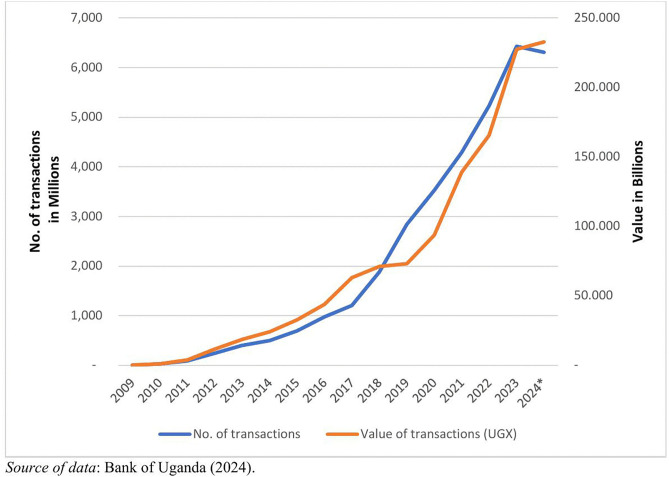
Trends in mobile money transaction volumes and values in Uganda. Source of data: Bank of Uganda (2024).

**Fig 2:**
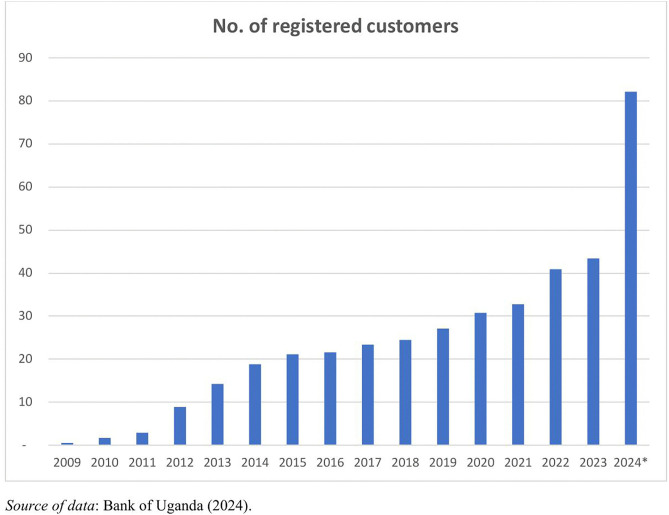
Trends in mobile money account registrations in Uganda. Source of data: Bank of Uganda (2024).

Since their introduction in 2009, mobile money services have profoundly transformed Uganda’s financial landscape, expanding access to expanding access and accelerating progress toward financial inclusion [6,7,35]. Their impact has been especially pronounced among micro, small, and medium-sized enterprises (SMEs) and the informal sector, which had long been excluded from formal finance. According to the Finscope Uganda 2023 survey, 65.9 percent of adults accessed financial services through mobile money, up from 54.4 percent in 2018, while roughly one in ten adults borrowed via mobile platforms [7]. Mobile money subscriptions increased from 14.2 million in 2013 to 43.4 million in 2024, and the total value of transactions rose from UGX 18.6 trillion to UGX 227.5 trillion (See Figs 1 and 2). This growth reflects the convenience and trust associated with platforms such as MTN Mobile Money and Airtel Money, which have become the default channels for payments, savings, and short-term credit.

The mobile money industry is private sector led but regulated by the Bank of Uganda (BoU) under the National Payment Systems Act, 2020, which granted mobile money full legal status and expanded the central bank’s supervisory mandate [35,36]. Earlier, the BoU’s 2013 Mobile Money Guidelines had required operators to partner with licensed financial institutions, but enforcement was limited, leaving telecoms largely accountable to the Uganda Communications Commission (UCC) [35,37]. The National Payment Systems Act, 2020 corrected this by conferring licensing authority on BoU, which issued Payment Service Provider (Class A) and Electronic Money Issuer licenses to MTN Mobile Money Uganda Ltd and Airtel Mobile Commerce (U) Ltd subsidiaries independent from their parent telecoms. While the new regime strengthened oversight, operational and consumer protection challenges persist. Cyber fraud, agent malpractice, and overlapping mandates between BoU and the Uganda Communications Commission (UCC) create compliance ambiguity [1,7,35]. In addition, weak rural network coverage and low digital literacy continue to limit financial inclusion [7,35].

Mobile money’s maturity has catalysed Uganda’s digital credit revolution. By 2024, mobile wallets had become the primary interface for short term borrowing, offering instant, unsecured loans to millions of users outside formal banking [1,7]. Telecom led products, most prominently MTN’s MoKash and Airtel’s Wewole, dominate the market. Borrowers initiate loans via USSD or mobile apps, creditworthiness is inferred algorithmically from transaction histories, funds are disbursed instantly, and repayments are automatically deducted from inflows [1,14]. For users, while this model has increased liquidity and reduced transaction frictions, pricing remains contentious [1,7,14,35]. MoKash loans range from UGX 3,000–1 million at a flat 9 percent fee per 30 days, while Wewole charges 6.75–15 percent per cycle with a 10 percent penalty on arrears [38–40]. Though simple to communicate, these flat fees translate into effective annualized costs exceeding 100 percent when rolled over, raising affordability concerns for low-income users.

The accessibility of digital credit has been accompanied by rising borrower risk [7,14]. There is increasing loan stacking, where borrowers take multiple concurrent loans across platforms lacking shared credit data. Automatic wallet deductions, while efficient for lenders, often exacerbate liquidity stress and reduce household resilience. Reports of aggressive recovery practices, frequent calls, SMS reminders, and social pressure, have raised concerns about conduct risk and consumer wellbeing. These developments underscore the tension between innovation driven financial access and the imperative of responsible lending. To address emerging risks, the Uganda Microfinance Regulatory Authority (UMRA) issued Digital Lending Guidelines in March 2024, setting 25 operational standards for responsible lending [41,42]. The framework requires digital lenders to obtain licenses, assess repayment capacity, disclose pricing transparently, and limit multiple borrowing and rollovers. Drawing on regulatory experiences from Kenya and Tanzania, the guidelines mark a significant step toward a unified supervisory regime. However, enforcement remains uneven in part because many unlicensed apps operate informally, data sharing mechanisms among lenders are weak, and consumer financial literacy is limited particularly in rural areas.

Overall, while technological innovation can democratize access to finance, it generates new policy challenges related to transparency, affordability, and consumer protection. In Uganda, mobile money providers continue to face security vulnerabilities, such as agent fraud, phishing, and unauthorized transactions, that erode user trust and strain operational integrity. In addition, while mobile based lending has improved liquidity and broadened inclusion, it has also exposed borrowers to high costs and opaque pricing structures. Sustaining this balance between innovation and protection will require stronger supervision, robust data governance and sharing frameworks, and ongoing collaboration among regulators, telecoms, and financial institutions to ensure that digital credit remains both inclusive and sustainable. Uganda’s experience illustrates both the promise and the fragility of digital credit in Sub-Saharan Africa. The period 2021–2024, characterized by post pandemic recovery, fintech innovation, and regulatory reform, offers a valuable context for studying these dynamics.

## Methodology

### Theoretical framework: An overview

Mobile loan disbursement by mobile money service providers is motivated by several economic, technological, and behavioural factors. These factors are grounded in various economic and financial theories that provide insight into lenders’ assessment of borrowers when determining loan amounts and managing financial risk. However, this study focuses on the theoretical frameworks of information asymmetry and adverse selection theory, credit rationing theory, and transaction economics to provide the theoretical foundation for the factors used to explain how mobile money service providers determine loan disbursement amounts.

In his seminal work, [43] developed the economic theory of asymmetric information, a crucial study focusing on adverse selection and moral hazard, with numerous follow-up studies and applications. Akerlof demonstrated that information asymmetries lead to adverse selection, where high-risk borrowers disproportionately dominate markets due to lenders’ imperfect risk assessment. In mobile money credit, this occurs when lenders lack complete borrower creditworthiness data, attracting more high-risk applicants. As a result, mobile lenders reduce loan sizes or increase interest rates for high-risk borrowers to counter this, while employing alternative credit scoring methods that utilise mobile transaction history, airtime usage, and digital footprints instead of traditional collateral.

Credit Rationing Theory was first introduced by [44], who argued that banks might not increase the interest rate charged, even in the face of excess demand for funds, as doing so might reduce their expected rate of return due to the increased probability of default. According to this theory, Lenders may not constantly adjust interest rates to compensate for risk because higher interest rates attract riskier borrowers. Instead, they may engage in credit rationing, meaning some borrowers receive less than requested or are denied loans despite being willing to pay high interest rates. Mobile money credit markets with asymmetric information may prefer credit rationing as a profit-maximising device and hence limit loan sizes for first-time borrowers, gradually increasing credit limits based on repayment behaviour and mobile money activity and/or excluding borrowers with low digital transaction activity from loans. Mobile lenders often start with small credit limits and increase them based on customers’ timely repayments [45,46].

The transaction cost theory is a key economic concept that focuses on the costs involved in economic exchanges [47]. It argues that transactions inherently come with costs, including negotiating contracts, overseeing performance, and resolving disputes. The theory suggests that the costs of organising transactions vary depending on whether they occur in a market or within a firm [48]. By comparing these transaction costs (or their indicators), the theory helps identify the most efficient way to conduct a transaction [49]. Williamson further explains that transactions involving specialised assets, frequent interactions, or high uncertainty incur greater costs due to opportunistic behaviour stemming from bounded rationality. Applying this theory to mobile lending reveals how digital platforms minimise transaction costs inherent in traditional lending, such as negotiation, credit verification, loan monitoring, and repayment collection. Mobile lending streamlines these processes through digital approvals, automated monitoring and electronic repayments, significantly reducing information costs via rapid data analysis. Additionally, features such as automated repayment tracking, digital reminders, and in app dispute resolution reduce reliance on physical interactions and manual administration. By improving access for remote users with minimal infrastructure demands, mobile lending aligns with the principles of transaction cost theory, offering a more efficient alternative that reduces friction, expenses, and barriers compared to conventional lending systems.

### Empirical model specification

This empirical study is grounded in the theoretical frameworks of information asymmetry and adverse selection theory, credit rationing theory and transaction economics, which explain lending decisions, borrower selection, and cost-efficiency considerations of transactions. Additionally, the study employs the Autoregressive Distributed Lag (ARDL) methodology for empirical estimation. The ARDL modelling framework is a popular econometric technique that instantaneously analyses both short-run and long-run relationships between variables in a time series framework. Proposed by [50] and [8], the ARDL approach is crucial when examining the dynamics of cointegration between variables that are integrated at different orders, i.e., I(0) and I(1) or a combination of both but not I(2) or higher [8]. The ARDL model offers distinct advantages over other cointegration techniques, including its robustness in small sample size modelling and its ability to address endogeneity, providing unbiased estimates and valid t-statistics, irrespective of the endogeneity of some regressors [8,50–53]. In general, ARDL models take the following form:


$$\begin{array}{c} {\text{Y}}_{t}=\alpha +\sum_{i=\text{1}}^{p}{\beta_{\text{i}}\text{Y}}_{t-i}+\sum_{j=\text{0}}^{q}{\gamma_{\text{j}}X}_{t-j}+\varepsilon_{t} \end{array}$$ (1)

where ${\text{Y}}_{t}$ is the dependent variable, α is a constant, ${\text{X}}_{t}$ represents the independent variables, p and q are the lags for Y and X, respectively, $\beta_{\text{i}}$ and $\gamma_{\text{j}}$are the coefficients and $\varepsilon_{t}$ is the error term. The specified empirical model for mobile loan disbursement is as follows:


$$\begin{array}{cc} {\Delta \text{l}LOAN}_{t}= & \beta_{\text{0}}+\sum_{k=\text{1}}^{n\text{1}}{\beta_{\text{1}k}\Delta \text{l}LOAN}_{t-k}+\sum_{k=\text{1}}^{n\text{2}}{\beta_{\text{2}k}\Delta l}_{t-k}\text{OLV}+\sum_{k=\text{1}}^{n\text{3}}{\beta_{\text{3}k}\Delta lINF}_{t-k}+\sum_{k=\text{1}}^{n\text{4}}{\beta_{\text{4}k}\Delta lINT}_{t-k}\\ & +\sum_{k=\text{1}}^{n\text{5}}{\beta_{\text{5}k}\Delta lCIEA}_{t-k}+\sum_{k=\text{1}}^{n\text{6}}{\beta_{\text{6}k}\Delta lAB}_{t-k}+\sum_{k=\text{1}}^{n\text{7}}{\beta_{\text{7}k}\Delta lCB}_{t-k}+\sum_{k=\text{1}}^{n\text{8}}{\beta_{\text{8}k}\Delta lFEES}_{t-k}\\ & +\sum_{k=\text{1}}^{n\text{9}}{\beta_{\text{9}k}\Delta lVOLT}_{t-k}+\sum_{k=\text{1}}^{n\text{1}\text{0}}{\beta_{\text{10}k}\Delta lATC}_{t-k}+\sum_{k=\text{1}}^{n\text{1}\text{1}}{\beta_{\text{11}k}\Delta lALR}_{t-k}\\ & +\gamma_{\text{0}}{lLOAN}_{t-k}+\gamma_{\text{1}}{lOLV}_{t-k}+\gamma_{\text{2}}{lINF}_{t-k}+\varepsilon_{t} \end{array}$$ (2)

where ∆ is a difference operator, 𝑙 denotes natural logarithm, $\beta_{\text{ik}}$ represents the short-run effect and $\gamma_{\text{ik}}$ represents the long-run effect, which are normalized by $\beta_{\text{0}}$. In Equation 2, LOAN is the average mobile loan disbursement value, OLV denotes Outstanding mobile Loans Value, INF is inflation, INT is the 91-day Treasury bill interest rate, CIEA denotes economic activity measured by the Composite Indicator of Economic Activity index, AB is Agents Balances, CB is Customer Balances, FEES means Mobile Money transaction fees, VOLT is the Volume of Transactions, ATC is Total airtime count. ALR is the average mobile loan repayment value. The study applies the ARDL estimation technique and EViews version 14.0 statistical software.

Mobile money providers are increasingly expanding into adjacent financial services, such as credit, savings, and insurance, with 44% of providers globally offering credit, making it the most widely available mobile money financial product as of June 2024 [54]. However, direct empirical evidence on the determinants of mobile money loan disbursements is scarce. Existing research shows that digital lending decisions rely heavily on behavioral data, especially repayment history and mobile phone usage, to predict repayment likelihood in the absence of formal credit records [14,16,46], with repayment history playing a central role in shaping future credit access and loan amounts [14,45,46]. Other signals, such as airtime purchases, are used in credit scoring models because they are linked to repayment probability and may reflect income stability [14,16,46], yet their effects on loan size decisions are not well established in empirical literature. Similarly, outstanding loan balances are used as indicators of existing debt and repayment burden, which can influence future borrowing limits [22,45], but their exact effect on loan disbursement amounts is not established in empirical literature. This study addresses this gap by empirically testing such relationships and, to the best of our knowledge, provides the first analysis of the determinants of mobile money loan disbursements in Sub Saharan Africa. The applied model specification combines behavioural indicators, platform level liquidity measures, transaction cost variables, and macro financial controls to explain variations in average mobile loan disbursement value. The contribution of the study lies in extending the digital credit literature beyond borrower selection, credit access, and repayment prediction to examine the determinants of mobile loan disbursement values.

According to the existing literature, economic activity, total airtime count, agent balances, transaction volumes, and average loan repayment value are expected to have a positive impact on average mobile loan disbursements [14]. In contrast, mobile money transaction fees are expected to have a negative impact on average mobile loan disbursements. Higher economic activity reflects improved income prospects, stronger business turnover, and enhanced repayment capacity. In such an environment, both the demand for working capital and consumption smoothing credit increases, while lenders perceive lower default risk. Consequently, we hypothesize that mobile lenders are more willing to extend larger or more frequent loans during periods of economic expansion. Total airtime count serves as a proxy for user engagement and digital footprint intensity within the mobile ecosystem. More frequent airtime purchases may signal income regularity, liquidity flows, and active platform usage, thereby improving assessed creditworthiness and increasing the likelihood of higher loan disbursements. Agent balances represent the liquidity capacity of the mobile money agent network. Since mobile loan disbursement ultimately depends on the operational liquidity of the ecosystem, stronger agent balances reduce settlement constraints and enhance the platform’s ability to intermediate funds efficiently. Greater agent liquidity therefore is expected to support credit expansion on the supply side. Transaction volumes reflect overall platform activity and the depth of financial intermediation within the mobile money system. High transaction intensity generates richer data for risk assessment and signals greater economic participation by users. From an information asymmetry perspective, more transaction data is expected to reduce uncertainty about borrower behavior, enabling lenders to extend larger loans with greater confidence. Average loan repayment value captures repayment performance and borrower discipline. Strong repayment patterns reduce perceived default risk and support dynamic limit increases under credit rationing models. In digital lending systems where credit limits are adjusted based on repayment history, improved repayment performance directly translates into higher subsequent disbursement levels [14,46]. In contrast, mobile money transaction fees are expected to have a negative effect on average loan disbursements. It is hypothesized that higher fees increase the effective cost of borrowing and transacting within the platform, potentially dampening loan demand. From the supply side, higher transaction costs may reduce net margins or discourage loan uptake, particularly for small, short tenor products where cost sensitivity is high. Thus, increases in transaction fees are likely to constrain average loan disbursement values.

However, *a priori* signs for outstanding loan values, inflation, 91-day interest rates, and customer balances are indeterminate. For instance, an increase in outstanding loans (i.e., available credit) may indicate a boost in consumer confidence in financial products, which generally encourages borrowing, including mobile loans. However, if loans grow too quickly or lead to higher defaults, lenders may become more cautious and reduce the issuance of new loans. Inflation may have mixed effects, as rising inflation can make it harder for borrowers to repay loans, prompting lenders to tighten their criteria. However, inflation driven by economic growth could boost income and employment, potentially offsetting the negative impact. A high 91-day Treasury bill rate increases borrowing costs and crowds out the mobile lending sector, as it competes with attractive government debt, which typically reduces demand for and supply of loans, including mobile money loans. However, mobile loan disbursements could rise if mobile lenders offer competitive rates to attract lower-risk borrowers, making them more attractive than bank loans despite the higher Treasury bill rate. Lastly, increased customer balances from higher income or remittances could lead to more borrowing for investment or consumption, resulting in more mobile loan disbursements. If customers choose to save rather than borrow, demand for loans may decline, resulting in reduced loan disbursements.

Following [8], the ARDL bounds test is used to determine whether a long-run relationship exists between variables. The null hypothesis of no cointegration is tested using the joint F-statistic as follows:


$$\begin{array}{c} H_{\text{0}}:\gamma_{\text{0}}=\gamma_{\text{1}}=\gamma_{\text{2}}=\gamma_{\text{3}}=\gamma_{\text{4}} \end{array}$$ (3a)

$$\begin{array}{c} H_{\text{1}}:\gamma_{\text{0}}\neq \gamma_{\text{1}}\neq \gamma_{\text{2}}\neq \gamma_{\text{3}}\neq \gamma_{\text{4}} \end{array}$$ (3b)

If the calculated F-statistic exceeds the upper bound or is below the lower bound, the null hypothesis is rejected, implying that there is cointegration. However, if the computed F-statistic is within the lower and upper bounds, the null hypothesis cannot be rejected, indicating no cointegration.

### Data sources and measurement of key variables

The study uses monthly data for the period of July 2021 to December 2024, a total of 42 observations. The choice of the sample period and data frequency is guided by data availability. Data on the variables of interest, namely average mobile loan disbursement value (LOAN), Outstanding Loans Value (OLV), inflation (INF), 91-day Treasury bill interest rate (INT), economic activity (CIEA), Agents Balances (AB), Customer Balances (CB), Mobile Money transaction fees (FEES), Volume of Transactions (VOLT), Total airtime count (ATC), and Average Loan Repayment (ALR) was obtained from Bank of Uganda’s database. LOAN is measured as the ratio of the total monthly disbursement of personal and business loans to the total number of loans disbursed in the same period by mobile lending providers. ALR is the ratio of the total value of monthly mobile Loan repayments to the total number of mobile Loan repayments in the same period. OLV represents the total unpaid loan balances owed by all mobile customers at the end of the month, reflecting their collective debt. ATC represents the number of times airtime was bought within a month. FEE captures the effects of mobile money transaction fee hikes on average mobile loan disbursement value by a dummy variable, which takes on the value of 1 following the hike in fees for Airtel’s mobile money services in Uganda in March 2024 and 0 otherwise. Inflation is measured as the first difference of the natural log of the consumer price index, where ${INF}_{t}=({lncpi}_{t}-{lncpi}_{t-\text{1}})\times \text{1}\text{0}\text{0}$.

Table 1 presents the descriptive statistics of the variables of interest employed in the analyses. The mean of average loan disbursement reveals small loans with low variation in loan disbursement values, suggesting that loan amounts are relatively small and consistent. The airtime count data shows less variation, implying that most values are close to the mean. However, outstanding loans and average loan repayment values exhibit higher variability. The high variability in loan repayment values may indicate irregular repayments, defaults, or differing borrower capacities to repay loans, which can also impact cash flow and liquidity for the lending institution. Meanwhile, inflation is low, with moderate variability, while economic conditions remain stable, exhibiting mild fluctuations as indicated by their respective means and standard deviations. Similarly, relatively low standard deviations in transaction volume, customer balances, the 91-day Treasury bill yield, and agent balances suggest that these variables are stable but subject to minor fluctuations.

**Table 1:** Descriptive statistics of variables employed in the analyses.

Variable	Description	Mean	Maximum	Minimum	Std. Dev.
LOAN	Value of average mobile loan disbursements	10.75	11.38	10.10	0.38
OLV	Outstanding loan value	24.89	26.60	21.84	1.29
INF	Inflation	1.43	2.37	0.62	0.54
INT	91-day Treasury bill yield	2.21	2.45	1.90	0.19
CIEA	Composite index of economic activity	5.06	5.14	4.97	0.05
AB	Agent’s balances	27.11	27.62	26.74	0.19
CB	Customer balances	27.26	28.48	27.03	0.24
FEES	Mobile money transaction fees	0.02	1.00	0.00	0.15
VOLT	Volume of transactions	20.04	20.49	19.67	0.20
ATC	Total airtime count	18.54	18.76	18.37	0.09
ALR	Average loan repayment	10.01	11.21	0.00	1.61

## Results and discussion

### Unit root and cointegration test results

The ARDL approach does not necessitate prior testing for the integration orders of the variables involved. However, within this framework, it is important to ensure that the variables are not integrated of order I(2), as this would invalidate the F-statistics and critical values derived by Pesaran et al. (2001). To determine the order of integration, we perform unit root tests, including the ADF, PP, and KPSS tests. Table 2 reports the unit test results, revealing a mixed order of integration between the variables, specifically I(0) and I(1). These results, with a mixed order of integration, support the use of the ARDL model.

**Table 2:** Results of unit root tests of variables employed in the analyses.

	Augmented Dicky–Fuller (ADF)		Phillips Peron (PP)		Kwiatkowski-Phillips-Schmidt-Shin (KPSS)		
Variable	Levels	1st Difference	Levels	1st Difference	Levels	1st Difference	Inference
Average Mobile Loan Disbursements-Value	−1.794	−5.196	−1.968	−5.207	0.550	0.045	I(1)
Outstanding Loans Value	−2.567	−5.617	−2.487	−6.737	0.550	0.190	I(1)
Inflation	−2.006	−3.718	−1.622	−3.926	0.161		I(1)/I(0)
91-day Treasury Bill Yield	−1.273	−6.074	−1.347	−6.117	0.515	0.102	I(1)
Composite Index of Economic Activity	0.079	−10.673	−0.155	−9.993	0.805	0.078	I(1)
Agents Balances	−3.389		−3.295		0.452		I(0)
Customer Balances	−3.863		−4.045		0.669	0.270	I(1)/I(0)
Volume of Transactions	1.625	−4.337	0.430	−25.549	0.824	0.225	I(1)
Total airtime count	0.222	−5.577	−1.658	−18.286	0.813	0.347	I(1)
Average Loan Repayment	−35.408		−20.345		0.164		I(0)
Asymptotic critical values							
Significance level	ADF		PP		KPSS		
1%	−3.601		−3.601		0.739		
5%	−2.935		−2.935		0.463		
10%	−2.606		−2.606		0.347		

The selection of the correct lag structure is vital for the accuracy of the model. The ARDL bounds testing approach requires that the optimal lag be chosen, assuming the residuals are not serially correlated. Pesaran et al. (2001) and later Narayan (2005) argue that the lag length should be sufficiently long to avoid serial correlation but not so long as to lead to over-parameterisation. We employed the Schwarz Information Criterion (SIC) automatic lag selection method to determine the appropriate lag length for each variable in the ARDL model, which resulted in a maximum lag length of 4 for both the dependent variable and the regressor. We employed Heteroscedasticity and Autocorrelation Consistent Covariance (HAC) estimators to correct for potential heteroscedasticity and autocorrelation, ensuring that the standard errors of the coefficients are adjusted without affecting the point estimates. Based on the SBC, the optimal model was ARDL (3,0,3), which is referred to as Model 1, and the analysis proceeded with this lag order. Based on Equation (2), the bounds test for cointegration was conducted using EVIEWS 14. Table 3 reports the bounds test results with an F-test statistic of 15.4, which exceeds the upper critical value of 6.6 at the 1% significance level. Consequently, the null hypothesis of no long-run equilibrium relationship between average mobile loan disbursement and the explanatory variables in Model 1 is rejected.

**Table 3:** ARDL bounds cointegration test results.

Dependent variable ^a^	F-Statistic for Case III Intercept with No Trend^b^	Conclusion
LOAN	6.875	Cointegration
OLV	1.532	No cointegration
INF	2.883	No cointegration

^a^The cointegrating vector includes the dependent variables of Average Mobile Loan disbursement value (LOAN), Outstanding Loans Value (OLV), and inflation (INF). Following Narayan (2005), the exact critical values for the F-statistics are computed for a small sample size of 42 observations.

^b^Critical values for the F-statistic at the 95 percent significance level, for case 3 (unrestricted intercept, no trend) with k = 2 and a sample size of 40, are 4.13 for the lower bound and 5.26 for the upper bound (see Narayan, 2005).

### Discussion of empirical results

Based on the cointegration test results, we estimated the long-run and short-run dynamics of average mobile loan disbursement. Table 4 reports the ARDL Conditional Error Correction results of Model 1. The long run and short run coefficients exhibit the expected signs, and the error correction term (ECT) is negative and statistically significant at the 1% level, reinforcing the existence of a long-run steady state relationship between average mobile loan disbursement and the explanatory variables. Table 4 indicates that, in the long run, the coefficients of the outstanding loan values and lagged inflation are of the expected sign but are statistically insignificant in influencing average mobile loan disbursement. This suggests that outstanding loan values and inflation do not influence average loan disbursement by mobile service providers in the long run, implying that their long term comovement does not translate into causal influence on mobile lending levels. The insignificance of inflation may reflect the structural features of Uganda’s digital credit ecosystem. Mobile loans are typically small, short-term facilities used primarily to smooth immediate liquidity constraints rather than finance long-term consumption or investment. In addition, lending decisions are largely automated and rely on customers’ mobile money transaction histories, airtime purchases, and repayment behavior rather than macroeconomic indicators. Consequently, mobile credit supply may respond more to real time digital activity within the mobile money ecosystem than to broader price dynamics [16,17].

**Table 4:** ARDL (3,0,3) model results for average mobile loan disbursement.

Variable	Coefficient
**Long run Estimates**	
Average mobile loan disbursement value (−1)	−0.766*** (−4.76)
Outstanding Loans Value	−0.023 (−0.58)
Inflation (−1)	0.041 (1.09)
**Short-run estimates**	
Δ Average mobile loan disbursement value (−1)	0.499*** (4.98)
Δ Average mobile loan disbursement value (−2)	0.298** (2.19)
Linear: Independent	
Δ Inflation	−0.047 (−0.22)
Δ Inflation (−1)	0.471** (2.41)
Δ Inflation (−2)	0.482 (1.50)
Deterministic	
Constant	−3.753
	(−0.50)
Fixed	
Δ 91-day Interest rate	−0.014
	(−0.04)
Δ Economic Activity	4.286
	(1.21)
Agents Balances	0.196
	(0.80)
Δ Customer Balances	0.047
	(0.64)
Mobile Money Transactions Fees	−0.222** (−2.52)

Δ Volume of Transactions	−3.522*** (−3.82)

Δ Total Airtime Count	3.194*** (3.43)
Average Loan Repayment	0.702*** (3.89)
Cointegrating Equation ECT(‐ 1)	−0.766*** (−4.74)
**Model Diagnostics**	
R-squared adjusted	0.83
Log-likelihood value	31.51
S.E. of regression	0.14
Schwarz Bayesian Criterion	−0.02
DW-statistic	2.38
Serial Correlation^1^	0.651[0.63]
F-statistic	12.163 [0.00]
Heteroscedasticity^2^	0.039 [1.00]
Normality^3^	1.748 [0.41]

^1^Breusch-Godfrey Lagrange multiplier test of residual serial correlation.

^2^ARCH test for Heteroskedasticity based on the regression of squared residuals on squared fitted values.

^3^Jarque-Bera Normality test based on a test of skewness and kurtosis of residuals. The values in parentheses are t-ratios, while probabilities are in brackets.

Table 4 presents the short-run dynamics of average mobile loan disbursements and indicates that current disbursement behaviour is strongly influenced by its past values. A 1 percent increase in the average loan disbursement value in the previous period (lag 1) leads to a 0.499 percent rise in the current period, while the second lag contributes an additional 0.298 percent increase. Both effects are statistically significant, confirming substantial positive persistence in mobile lending behaviour. This finding suggests that past disbursements continue to exert a strong influence on current lending decisions, although the effect diminishes slightly at the second lag, implying that the influence of prior lending activity persists for at least two months.

Table 4 shows that current inflation does not have a statistically significant effect on average mobile loan disbursements in the short run. However, the lagged inflation variable has a moderate and positive coefficient, indicating that a 1 percent rise in inflation in the previous period is associated with a 0.471 percent increase in current average loan disbursement values. This suggests that periods of rising prices may coincide with higher nominal lending volumes as borrowers adjust loan demand to meet increased transaction and consumption costs. This pattern suggests that mobile lenders may not respond immediately to changes in the price level; however, inflation affects repayment capacity, disposable income, and risk assessment with a delay and as such automated scoring models may update credit limits gradually as inflation influences repayment performance and liquidity conditions over time. The lagged significance therefore reflects adaptive risk adjustment rather than instantaneous macro sensitivity.

By contrast, changes in the 91-day Treasury bill yield, Composite Index of Economic Activity (CIEA), agent balances, and customer balances are statistically insignificant, although their coefficients carry the theoretically expected signs. This implies that, in the short run, these macroeconomic and institutional factors exert limited influence on mobile money loan disbursements, underscoring the market’s relative insulation from short term monetary policy and aggregate output fluctuations. In Uganda, mobile money lenders do not intermediate deposits in the same manner as commercial banks, nor are their funding structures tightly linked to sovereign yield movements. As a result, short-term fluctuations in Treasury bill rates may not immediately affect the cost structures or credit allocation decisions of mobile network operators. This attenuated interest rate sensitivity is consistent with the institutional dominance of MNO operated ecosystems over bank driven credit intermediation in the digital microcredit segment. Similarly, the insignificance composite index of economic activity may reflect the functional role of mobile loans within Uganda’s financial landscape. Digital credit is frequently used for short-term liquidity management, emergency consumption smoothing, and micro working capital rather than longer-term investment projects that are closely aligned with aggregate business cycles and as such in a market where informal activity remains substantial and high frequency cash flow fluctuations dominate borrowing decisions, real time transactional behaviour may carry greater explanatory power than aggregate macroeconomic indicators. While agent balances and customer balances proxy platform liquidity and wallet level funds availability, their insignificance suggests that mobile money lenders prioritize behavioural and transactional data over snapshot balances when assessing creditworthiness.

The results indicate that Total airtime count and Average loan repayment exert statistically significant and positive effects on Average mobile loan disbursements. A 1 percent increase in total airtime count and average loan repayment corresponds to respective increases of 3.19 percent and 0.702 percent in average loan disbursement values. The strong positive association between airtime usage and loan disbursements suggests that mobile lenders employ airtime purchases as behavioural proxies for digital credit scoring. Frequent airtime purchases reflect regular income flows and consistent financial activity within the mobile money ecosystem, signalling a borrower’s creditworthiness, making these customers more eligible for more loan disbursements. Similarly, the positive effect of average loan repayment implies that consistent repayment performance enhances lender confidence, supports liquidity recycling, and promotes further loan issuance. These behavioural variables thus function as informational substitutes for traditional credit histories in data-scarce environments, reinforcing the role of digital footprints in mitigating information asymmetry and sustaining the expansion of mobile based credit markets [1,14].

The results show that mobile money transaction volume and transaction fees have statistically significant negative effects on average mobile loan disbursements. A 1 percent increase in transaction volume and transaction fees is associated with respective decreases of 3.52 percent and 0.222 percent in average loan disbursement values. These results imply that higher user costs and intensified transaction activity constrain credit expansion in the short run. Elevated fees discourage borrowing by raising the effective cost of credit, while high transaction volumes likely increase operational expenses for mobile lenders, prompting them to tighten lending conditions or adjust pricing to preserve margins. The adverse effect of transaction fees also reflects a behavioural response by borrowers: when costs rise or pricing becomes opaque, loan demand declines. For example, in March 2024, Airtel’s mobile money services in Uganda quietly hiked the cost of borrowing without informing customers. This failure to provide consumers with a clear fee structure and pricing information not only represents a critical consumer protection challenge but also directly harms consumers, potentially leading to a breakdown in trust between them and their financial service providers, and resulting in reduced platform service usage [55].

The impact of high transaction volume on mobile lenders’ fees and lending practices was found to be statistically significant. This is because high transaction volumes often lead to increased operational costs for lenders, which they may pass on to borrowers through higher fees and more stringent lending terms. This may discourage borrowers from obtaining new loans. Mobile lenders may increase interest rates to cover the costs associated with processing a high volume of transactions, which makes loans more expensive for borrowers, particularly those already struggling financially. Moreover, high transaction volumes can attract regulatory scrutiny, leading to more stringent oversight and enforcement of anti-predatory lending laws to safeguard borrowers from unfair lending practices. Further, mobile lenders may use transaction volumes as market indicators in their risk assessment process, with unusually high trading volumes signalling market volatility or uncertainty, which could lead lenders to be more cautious about loan disbursements.

The negative and sizeable magnitude of the error correction term (ECT) indicates a rapid speed of adjustment toward equilibrium, with approximately 76.6 percent of any short run deviation corrected within a month. This implies that the mobile money loan disbursement system responds swiftly to shocks, realigning with its long run trajectory in roughly two months. Such a high adjustment rate suggests that Uganda’s digital credit market is highly adaptive, with lenders and borrowers quickly recalibrating behaviour in response to short term imbalances or market fluctuations.

### Model specification and robustness test results

Table 4 reports the model diagnostic tests used to ascertain the robustness of the estimated model. The results reveal that the ARDL (3,0,3) model passes all the diagnostic tests, suggesting that this model can be reliable for policy-making and statistical inference. The test statistics for serial correlation and heteroscedasticity, at the 5% significance level, led to the acceptance of the null hypothesis of no serial correlation and homoscedasticity. In addition, the test for the overall significance of the estimated models depends on the significance of the F-statistic and the F-statistic values for both models are highly significant at a 1% level of significance, an indication that all the explanatory variables in the estimated models are jointly statistically significant in explaining the changes in average mobile loan disbursements.

**Fig 3:**
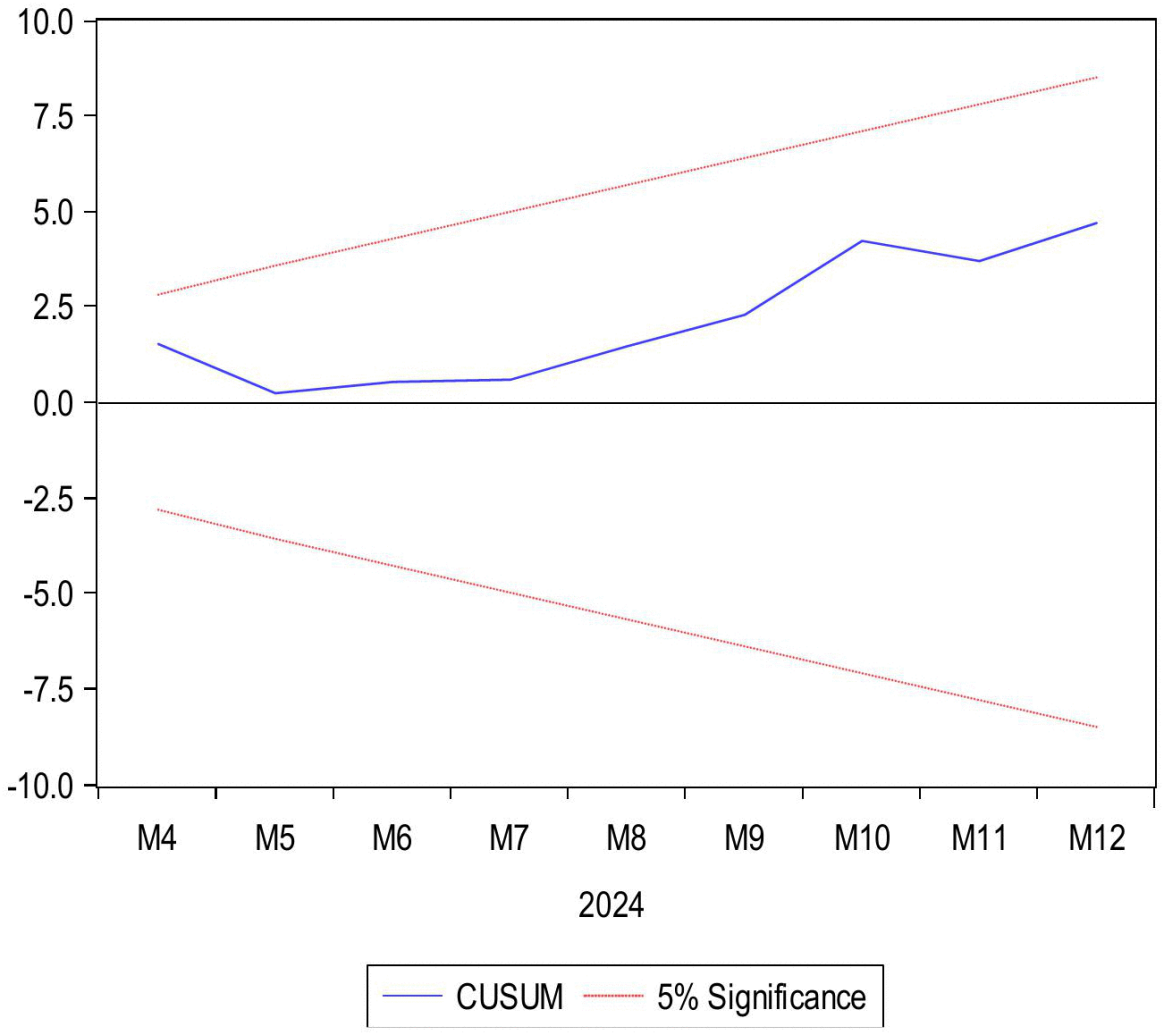
Plot of CUSUM test for the estimated ARDL model.

**Fig 4:**
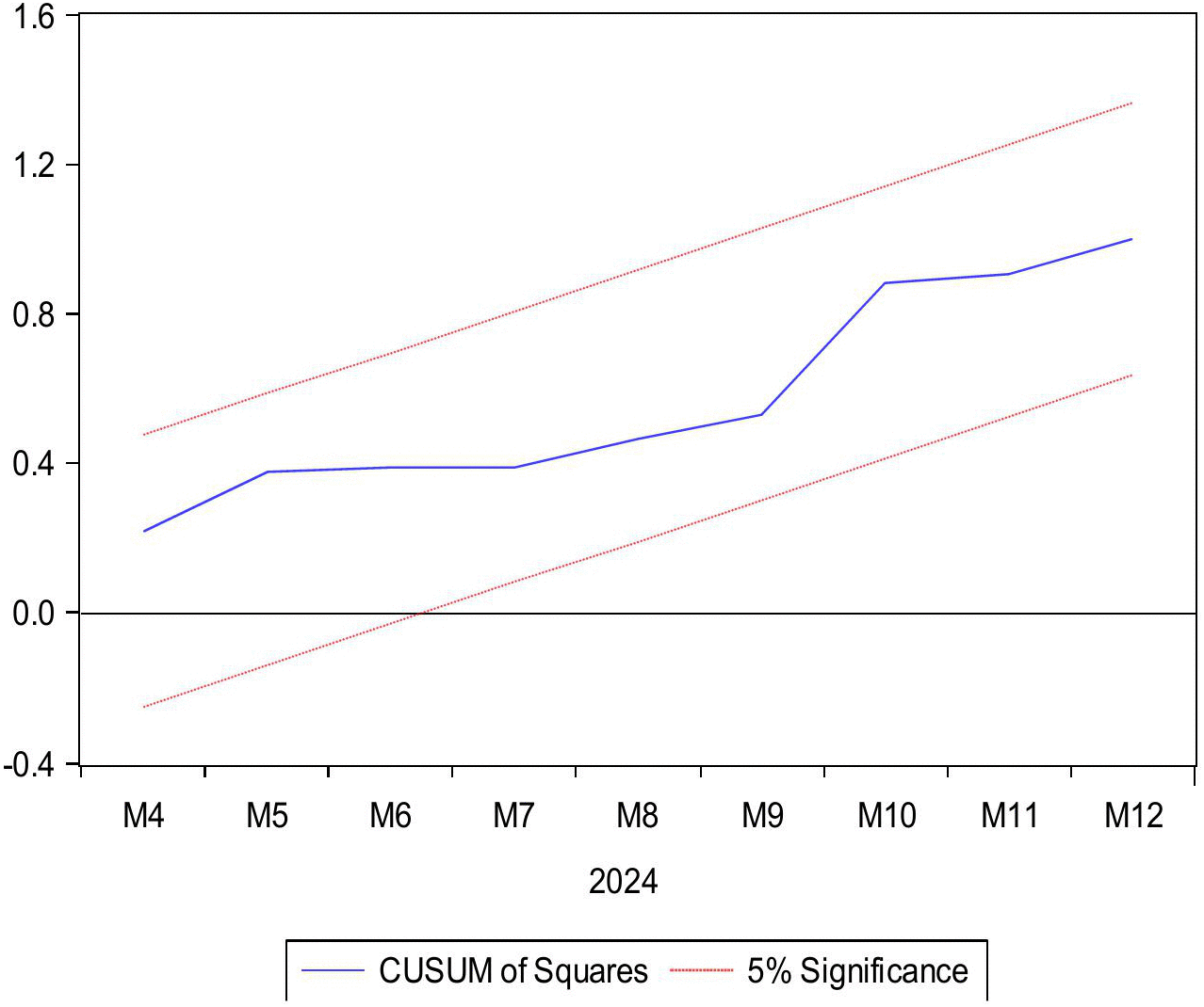
Plot of CUSUMSQ test for the estimated ARDL model.

Figs 3 and 4 shows the results for parameter stability, with both the CUSUM test and the CUSUMSQ test indicating parameter stability. The test results indicate parameter stability when the cumulative sum falls within the 5% critical lines. Thus, the results of the Cumulative sum tests in this study suggest that the ARDL model is stable, meaning that the estimators accurately measure the relationship between the cointegrated variables.

## Conclusion and policy implications

In the aftermath of the COVID-19 pandemic, Uganda experienced a sharp rise in mobile money usage and digital credit adoption, positioning it as a stellar case for examining the determinants of average mobile money loan disbursements. Using monthly data from July 2021 to December 2024, this study applies the Autoregressive Distributed Lag (ARDL) cointegration framework of [8] to capture both short run dynamics and long-run relationships among institutional, macroeconomic, and behavioural variables influencing lending activity. Cointegration test results confirm a stable long run association among average loan disbursements, outstanding loan values, and inflation, indicating that these variables move together over time. However, in the estimated long run model, neither outstanding loans nor inflation significantly affects disbursement levels, implying that their long term comovement does not translate into direct causal impact. In the short run, past disbursements exert a strong positive effect on current average loan disbursement values, suggesting persistent lending patterns in Uganda’s mobile credit market. Lagged inflation exerts a modest positive influence, which may reflect temporary nominal adjustments in loan values as borrowers adjust loan demand to meet increased transaction and consumption costs. By contrast, the 91-day Treasury bill yield and Composite Index of Economic Activity are statistically insignificant, underscoring the limited role of monetary policy and aggregate output conditions in shaping mobile lending behaviour during the study period.

Behavioural indicators remain highly informative. Total airtime count and Average Loan Repayment positively affect loan disbursements, suggesting that mobile lenders interpret frequent airtime usage and timely repayments as reliable signals of liquidity and creditworthiness. Conversely, high Volume of Transactions and elevated Mobile Money transaction fees significantly reduce average loan disbursements, indicating that higher user costs discourage borrowing and constrain credit expansion. The negative and significant error-correction term (ECT) confirms rapid adjustment toward long run equilibrium, with deviations corrected within one to two months, demonstrating the adaptive nature of Uganda’s mobile lending system.

From a policy perspective, these findings highlight the need for regulatory vigilance to balance innovation with financial stability. Regulators should monitor fee structures, promote transparent lending practices, and strengthen consumer protection frameworks. Moreover, reliance on behavioural data for credit scoring calls for stronger data governance standards to safeguard user privacy. While this study provides novel empirical evidence on the supply side drivers of digital credit in Uganda, two limitations merit acknowledgment. The use of aggregate monthly data may mask heterogeneity in borrower characteristics, and the relatively short sample period may not fully capture structural shifts in the evolving digital finance landscape. Future research incorporating borrower level data could further illuminate the welfare implications of digital lending.

Uganda’s post pandemic experience ultimately reflects both the promise and fragility of digital credit markets in frontier economies, where technological innovation can broaden access to finance, but without prudent regulation, it risks amplifying costs and consumer vulnerabilities.
